# Single-visit versus two-visit endodontic treatment for postoperative pain in asymptomatic non-vital mandibular premolars

**DOI:** 10.6026/973206300221302

**Published:** 2026-03-31

**Authors:** Pooja Bala, Poonam Bogra, Swati Chhabra, Nikita Goyal, Niharika Soni, Himani Arora, Surinder Sachdeva

**Affiliations:** 1 Department of Conservative Dentistry and Endodontics, J.N. Kapoor DAV Centenary Dental College, Yamunanagar, Haryana, India; 2 Department of Conservative Dentistry and Endodontics, M.M College of Dental Sciences and Research, MM (Deemed to be University), Mullana, Ambala, India; 3 Department of Periodontics, M.M College of Dental Sciences and Research, Maharishi Markandeshwar (Deemed to be University), Mullana, Ambala, Haryana, India

**Keywords:** Calcium hydroxide, intracanal medicament, iodine potassium iodide (IKI), postoperative pain, single-visit root canal, two-visit root canal

## Abstract

Postoperative pain is one of the primary problems in endodontic treatment and the ideal protocol for managing non-vital teeth through single-visit or multiple-visit endodontic treatment remains unsettled. Therefore, it is of interest to compare postoperative pain following single-visit endodontic therapy using 5% Iodine Potassium Iodide (IKI) as a final irrigant with that is following two-visit treatment using calcium hydroxide as an intracanal medicament. Seventy asymptomatic non-vital mandibular premolars were randomly assigned to two groups. Postoperative pain was assessed using Visual Analog Scale at 24, 48, 72 hours and one week after obturation. Both groups showed a similar trend of pain reduction over time. The mean pain scores were slightly lower in the IKI group at all intervals, though the differences were not statistically significant.

## Background:

Root canal treatment (RCT) is a common dental procedure primarily indicated for irreversible pulpitis and pulp necrosis resulting from caries or dental trauma [1]. The procedure involves the removal of inflamed or necrotic pulp tissue and elimination of bacteria from the root canal system through mechanical instrumentation combined with chemical irrigation as for thorough cleaning, decontamination, and shaping of the canals eeffective chemo-mechanical preparation is essential [2]. While mechanical instrumentation plays a key role in reducing the microbial load, it fails to contact approximately 30-40% of the canal walls [3]. Therefore, irrigating solutions are crucial, as they enhance disinfection by aiding in the removal of microorganisms, necrotic tissue, and dentinal debris during and after instrumentation [4]. Calcium hydroxide is a widely used intracanal medicament due to its strong antibacterial activity, attributed to its high pH (12.5-12.8) and low solubility and can be applied for 1-2 weeks between appointments [5, 6]. Ideally, if a fast-acting antimicrobial agent could disinfect canals within minutes, it would eliminate the need for inter-appointment dressings, making single-visit root canal treatment more feasible [7, 8, 9-10]. Iodine potassium iodide (2-5%), one such medicament, when used as a final irrigant for 5-15 minutes is effective anti-bacterial, hypoallergenic and minimal cytotoxic [11]. Therefore, it is of interest to compare postoperative pain in asymptomatic non-vital mandibular premolars treated in a single visit using 5% IKI as a final irrigant versus a two-visit approach using calcium hydroxide as an intracanal medicament

## Materials and Methodology:

The present randomized controlled study was conducted in the Department of Conservative Dentistry and Endodontics after obtaining approval from the Institutional Ethical Committee (Ref. No. IEC: F/EC/24/23/0218). Written informed consent was taken. The study included systemically healthy patients (ASA I and ASA II) aged 18-40 years with asymptomatic non-vital mandibular premolars exhibiting fully formed apices and no anatomical variations. Teeth presenting with pus discharge, sinus tract, internal or external resorption, periodontal pockets exceeding 4 mm, mobility greater than grade I, or periapical radiolucency of ≥3 mm were excluded from the study. The sample size was calculated using the SPSS Statistical software 21.0 version. The power of the study was taken to be 90% and Confidence Interval (C.I.) of 95% was taken. The sample size calculated was a minimum of 29 per group but to compensate the dropout of 20%, a total of 35 per group were included in the study. The samples were divided into two groups Group I (n=35) and Group II (n=35).

## Clinical procedure:

Local anaesthesia was administered and rubber dam isolation was done. After standardized access cavity preparation, shaping was done with Hyflex CM files followed by irrigation with of saline (2 ml), 17% EDTA (2 ml), and finally with saline. Canal was dried with paper point to remove excess irrigant. In Group I (single-visit) - final irrigation was performed with 5% IKI for 10 minutes before obturation. Group II (two-visit) - Calcium hydroxide paste (in propylene glycol) was placed for two weeks as an intracanal medicament before obturation. Postoperative pain was assessed at 24, 48, 72 hours, and one week after obturation using Visual Analog Scale.

## Statistical analysis:

Data were analyzed using Statistical Package for Social Sciences (SPSS). Categorical outcome variables were analyzed by Chi-square test.

## Results:

No statistically significant difference (p > 0.05) was observed between the groups at any interval. The mean postoperative pain levels were higher at 24 hours in both groups and decreased progressively over time. By 72 hours, all patients in Group I and most in Group II were pain-free (Table 1 - see PDF). However, the mean pain score was slightly lower in Group I when compared with Group II at all-time intervals.

## Discussion:

Postoperative pain after root canal treatment is a multifactorial outcome; reported incidence of moderate to severe postoperative pain within 24 hours ranges between 47% and 60% [12]. Mandibular premolars were selected due to their narrow mesiodistal dimensions, apical curvatures, and frequent canal ramifications, which make debridement difficult and may increase the likelihood of treatment failure [13]. In this randomized controlled clinical study postoperative pain in asymptomatic non-vital mandibular premolars treated with a single-visit endodontic protocol using 5% iodine potassium iodide (IKI) as a final irrigant and a conventional two-visit approach employing calcium hydroxide as an intracanal medicament exhibited mild postoperative pain during the first 48 hours, which gradually subsided, and all patients were asymptomatic after one week. The study's results align with findings from Al-Negrish *et al.* and Vieyra *et al.* who also concluded no significant difference in postoperative pain between single- and multiple-visit treatments [14, 15]. However, Albashaireh *et al.* reported higher postoperative pain in multi-visit cases lacking intracanal medicaments, emphasizing the importance of chemical disinfection in pain reduction [16]. While there was no statistically significant difference between the two groups, the mean pain score was relatively lower in Group I when compared with Group II. The probable reason may be due to immediate obturation, preventing interappointment contamination and the potent antimicrobial action of IKI [8]. When used after NaOCl and EDTA, IKI enhances dentinal permeability, penetrating up to 1000 µm into dentinal tubules, which results in superior antimicrobial efficacy [17]. Calcium hydroxide remains the gold standard intracanal medicament but for maximal effect, it requires two to three weeks of placement to allow ionic diffusion through dentin and periapical tissues [18]. Despite its efficacy, longer treatment time, potential for interappointment leakage and reduced patient compliance are notable limitations, which could explain the slightly higher postoperative pain in the two-visit group [19, 20]. Thus, 5% IKI used as a final irrigant for 10 minutes can be considered clinically comparable to a two-week calcium hydroxide dressing in controlling postoperative pain and ensuring patient comfort in asymptomatic non-vital mandibular premolars

## Conclusion:

Both single-visit IKI and two-visit calcium hydroxide protocols produced comparable postoperative pain outcomes. Therefore, in asymptomatic non-vital mandibular premolars both approaches are acceptable.
